# A trial of ciprofloxacin vs aminoglycoside-ciprofloxacin for bubonic plague

**DOI:** 10.1056/NEJMoa2413772

**Published:** 2025-08-07

**Authors:** Rindra Vatosoa Randremanana, Mihaja Raberahona, Josephine Bourner, Minoarisoa Rajerison, Tansy Edwards, Ravaka Randriamparany, Tsinjo Fehizoro Razafindratsinana, Lisy Hanitra Razananaivo, Gabriella Zadonirina, Théodora Mayouya-Gamana, Alex Paddy Abdel Salam, Reziky Tiandraza Mangahasimbola, Voahangy Andrianaivoarimanana, Elise Pesonel, Rivonirina Andry Rakotoarivelo, Mamy Jean de Dieu Randria, Peter Horby, Piero Olliaro

**Affiliations:** 1 Institut Pasteur de Madagascar, Antananarivo, Madagascar; 2 CHU Joseph Raseta Befelatanana, Antananarivo, Madagascar; 3 Équipe de Recherche Clinique en Maladies Infectieuses Madagascar; 4 Centre d’Infectiologie Charles Mérieux, Université d’Antananarivo, Madagascar; 5 ISARIC, Pandemic Sciences Institute, Nuffield Department of Medicine, University of Oxford, UK; 6 International Statistics and Epidemiology Group, London School of Hygiene and Tropical Medicine, London, UK; 7 The Biodiscovery Institute, The University of Nottingham, United Kingdom; 8 CHU Tambohobe Fianarantsoa, Madagascar, University of Fianarantsoa, Madagascar

## Abstract

**Background:**

Plague is a high-consequence infectious disease of epidemic potential. Current treatment guidelines are based on weak evidence.

**Methods:**

IMASOY enrolled individuals of any age and sex (excluding pregnancy) with clinically suspected bubonic plague during 2020-2024 at 47 sites in 11 districts in Madagascar.

We compared two regimens included in the country’s guidelines using an open-label non-inferiority design: ten-day oral ciprofloxacin (intervention) vs. three-day injectable aminoglycoside followed by seven-day oral ciprofloxacin (control). The primary endpoint was treatment failure on day eleven defined as death, fever, secondary pneumonic plague, alternative or prolonged plague treatment. For non-inferiority in the primary intent-to-treat infected population (ITTI: laboratory confirmed/probable infections), the 97.5% upper bound of the confidence interval around the risk difference was to be <15%.

**Results:**

Of 933 patients screened, 450 patients with suspected bubonic plague were enrolled and randomised, with 220 confirmed and 2 probable infections; 53.2% male, median (range) age 14 years (2-72). Ciprofloxacin monotherapy was non-inferior to control: 9.0% (10/111) vs. 8.1% (9/111) treatment failures, 0.9% difference (95% confidence interval –6.0 to 7.8%). Non-inferiority was consistent in other pre-specified analysis populations. Five and four patients died respectively, three patients per arm developed secondary pneumonic plague. Similar percentages of participants experienced serious adverse events (ITTI: intervention 7.2%; control 5.4%) and adverse events (ITTI: 18.0% and 18.9%, respectively).

**Conclusions:**

Ciprofloxacin oral monotherapy for 10 days is non-inferior to aminoglycoside-ciprofloxacin sequential combination for treating bubonic plague.

(Funded by UK Foreign, Commonwealth and Development Office and other; **Trial registration number**: NCT04110340)

Plague is a zoonosis caused by *Yersinia pestis*, a Gram-negative bacterium, maintained in nature through transmission between fleas (e.g., *Xenopsylla cheopis*) and their rodent hosts.^1,2^ Human infection occurs following a bite from an infected flea, direct contact with infectious body fluids or tissues of an infected human or animal, or inhalation of infectious respiratory droplets. Human plague manifests clinically in bubonic, pneumonic or septicaemic forms. Bubonic plague, the most common form, is characterised by swollen, painful lymph nodes – ‘buboes’ – accompanied by non-specific symptoms including fever, headache, altered mental status, chills and malaise.^3^

Case fatality rates (CFR) of bubonic plague were over 75% in the pre-antibiotic era.^4^ Current World Health Organization (WHO) estimates are between 17-26%^5^, whereas a systematic review found an overall CFR of 15%, with higher rates in low-income settings (17%) and lower rates in clinical research settings (5%).^3,6^ Since the first documented human cases 4,900 years ago,^7^ *Y. pestis* lineages have spread causing devastating epidemics and pandemics throughout human history.^8,9^ The number of cases has been steadily declining over past century, with 248 cases of plague reported to the WHO in 2018 – 98% from Madagascar and the Democratic Republic of Congo.^10^

However, plague remains a high-consequence disease due to the widespread rodent reservoir^11^ and epidemic potential, including the potential risk for bioterrorism via deliberate release. ^12^

Plague is endemic to Madagascar with annual peaks generally occurring between August and April, mostly during the rainy season. However, in 2017, an urban epidemic caused 418 confirmed or probable cases of pneumonic and 139 of bubonic plague in four months, with a CFR of 25% and 24% among confirmed cases, respectively.^13^ During 2018-2024, a total of 1134 suspected bubonic plague cases were notified to the plague central laboratory, hosted by Institut Pasteur de Madagascar (IPM), of which 598 (53%) were confirmed/probable.

While several treatment regimens for plague are recommended in treatment guidelines ^5,14^, they are supported by weak evidence, with no pivotal randomised controlled trials successfully completed.^6,15^ Some regimens are approved by the United States Food and Drugs Administration (US FDA) under the ‘animal efficacy rule’^16^ – streptomycin, doxycycline and other tetracyclines; and the fluoroquinolones ciprofloxacin, levofloxacin, and moxifloxacin – others, though not FDA-approved, are generally considered effective – gentamicin, chloramphenicol, and trimethoprim-sulfamethoxazole. For all plague treatments there are little or no human efficacy data.^17^

Significant challenges hamper the conduct of plague treatment trials: logistical – small number of cases often occurring in remote rural areas; accessibility; security and social instability in some plague-endemic areas; and methodological – definition of an agreed endpoint of cure or improvement.

The 2018 guidelines for the treatment of bubonic plague in Madagascar recommend for adults a 3-day course of injectable aminoglycoside (first-line streptomycin, second-line gentamicin) followed by a 7-day course of oral ciprofloxacin, and a 10-day course of oral ciprofloxacin as third-line option; this treatment is the first-line choice for children under 15 years-old (Supplementary Appendix S1 Table).

Lack of higher quality evidence supporting treatment regimens and shortcomings of aminoglycosides (requiring injections, toxicity, poor intracellular penetration) prompted us to design a randomised controlled trial (RCT) comparing these two regimens.^18^

## Methods

IMASOY (NCT04110340) is a multi-centre open-label, randomised controlled trial evaluating the non-inferiority of a ciprofloxacin monotherapy against aminoglycoside-ciprofloxacin sequential combination for bubonic plague in Madagascar.

The trial was approved by Oxford Tropical Research Ethics Committee (45-18), Comité d’Ethique et de Recherche Biomédicale à Madagascar (Authorisation N°116-MSANP/CERBM dated 10/09/2018)), and the London School of Hygiene and Tropical Medicine (17911). Further details about the trial design can be found in the Supplementary Appendix and in the protocol publications and at nejm.org. ^19,20^

### Participants

Recruitment took place in health centres and hospitals in plague-endemic districts in Madagascar during the annual plague transmission season – typically from August to April.

Patients with clinically suspected bubonic plague, of any age and sex, were eligible if they had a recent onset or history of fever, one or more buboes, and residence in or travel to a plague endemic area within 14 days before symptom onset. Reasons for exclusion were a known allergy to aminoglycosides or fluoroquinolones, tendinitis, myasthenia gravis, theophylline or warfarin use, or had already received treatment for plague in the preceding three months, or pregnancy.

Cases definition was according to the international case definition for plague. ^2^ A confirmed case required a positive qPCR or culture on D1 sample or an anti-F1 IgG seroconversion in paired serum samples or a four-fold increase in antibody titre up to Day 21.

A probable case was defined as only a positive F1-antigen rapid test conducted at the central laboratory or a single anti-F1 serology positive without evidence of previous *Y. pestis* infection or vaccination. All other suspected patients enrolled were subsequently considered non-cases.

All diagnostics were laboratory-developed tests designed, manufactured and routinely used at the Central Plague Laboratory (CPL), hosted at Institut Pasteur of Madagascar (IPM), and WHO Collaborating Centre for plague.

### Interventions

The control arm was an aminoglycoside followed by ciprofloxacin. Streptomycin was given at 1g twice daily to adults (15mg/kg twice daily to children), gentamicin at 2.5mg/kg IV for three days, followed by ciprofloxacin at 500mg orally twice daily to adults (15mg/kg twice daily, not to exceed 500mg per dose to children) for an additional seven days. The intervention arm was ciprofloxacin monotherapy for ten days at the same daily doses.

### Outcomes

The primary efficacy outcome was treatment failure assessed on Day (D11) using a composite outcome defined as death, fever, development of secondary pneumonic plague or receipt of alternative or additional treatment for plague up to and including D11.

A secondary efficacy outcome was treatment failure as above with an additional component of less than a 25% reduction in bubo size at D11.

Other descriptive secondary outcomes were; proportion of patients with fever at D4; proportion of patients who developed secondary pneumonic plague; proportion of patients with a bubo pain score < 3 at D4 and D11; mean percentage change in bubo size at D4 and D11, proportion of patients experiencing a serious adverse event on or before D4, D11 and D21; and proportion of patients who adhered to the study treatment schedule.

### Sample size

Assuming 90% of individuals receiving an aminoglycoside plus ciprofloxacin would meet the primary endpoint of therapeutic response on D11 (10% treatment failure), 190 confirmed or probable bubonic plague cases (95 per group) were required to have 90% power to demonstrate the non-inferiority of a ciprofloxacin monotherapy, with a 15% non-inferiority margin and a one-sided alpha of 2.5% and allowing for 10% loss to follow-up.

### Randomisation and blinding

Patients were randomised following consent by the trial team at participating sites with a 1:1 allocation ratio, using a computer-generated randomisation sequence with random block sizes generated from a master list by the trial statistician and stratified by health facility. Blinding of patients and the trial team to treatment allocation was not possible due to the different treatment administration routes.

### Statistical methods

The primary analysis population consists of confirmed and probable cases of bubonic plague who were randomised and received the trial drugs (intention-to-treat infected, ITTI). Other pre-specified analysis populations for the primary endpoint were the ITT (all), per-protocol (all) and per-protocol infected.

Baseline data are summarised overall for the ITT and ITTI populations and by arm in the ITTI population, using number, percent, median and range as appropriate.

The frequency of each component of the composite primary endpoint was reported as number and percent by arm. A corresponding 95% confidence interval (CI) was reported for the risk of overall treatment failure per arm and the risk difference in failure between arms. For non-inferiority of ciprofloxacin against control, the upper bound of 95% CI around the risk difference was to be less than 15%.

The primary analysis of the primary efficacy outcome was adjusted for trial site using robust standard errors in a generalised linear binomial model with an identity link function. Unadjusted results are also presented. Adjusted and unadjusted between-arm non-inferiority comparisons were repeated for the primary efficacy endpoint in the ITT, PP and PPI analysis populations and the secondary efficacy outcome incorporating bubo size in the ITTI analysis population. Pre-specified sensitivity analyses are described in the Supplementary Appendix.

Descriptive secondary outcomes were summarised by arm in the ITTI population, using number, percent, median and range as appropriate.

Safety data were summarised as the number and percent of patients experiencing at least one serious adverse event, at least one adverse event and at least one adverse drug reaction (ADR), up to D21, in both the ITT and ITTI populations. Events were considered as an ADR if the causality assessment determined a possible, probable or certain relationship to the trial drugs. Incidence of adverse drug reactions is reported by the Medical Dictionary for Regulatory Activities (MedDRA; v27.1) preferred terms, organised by system organ class.

## Results

**Figure 1:**
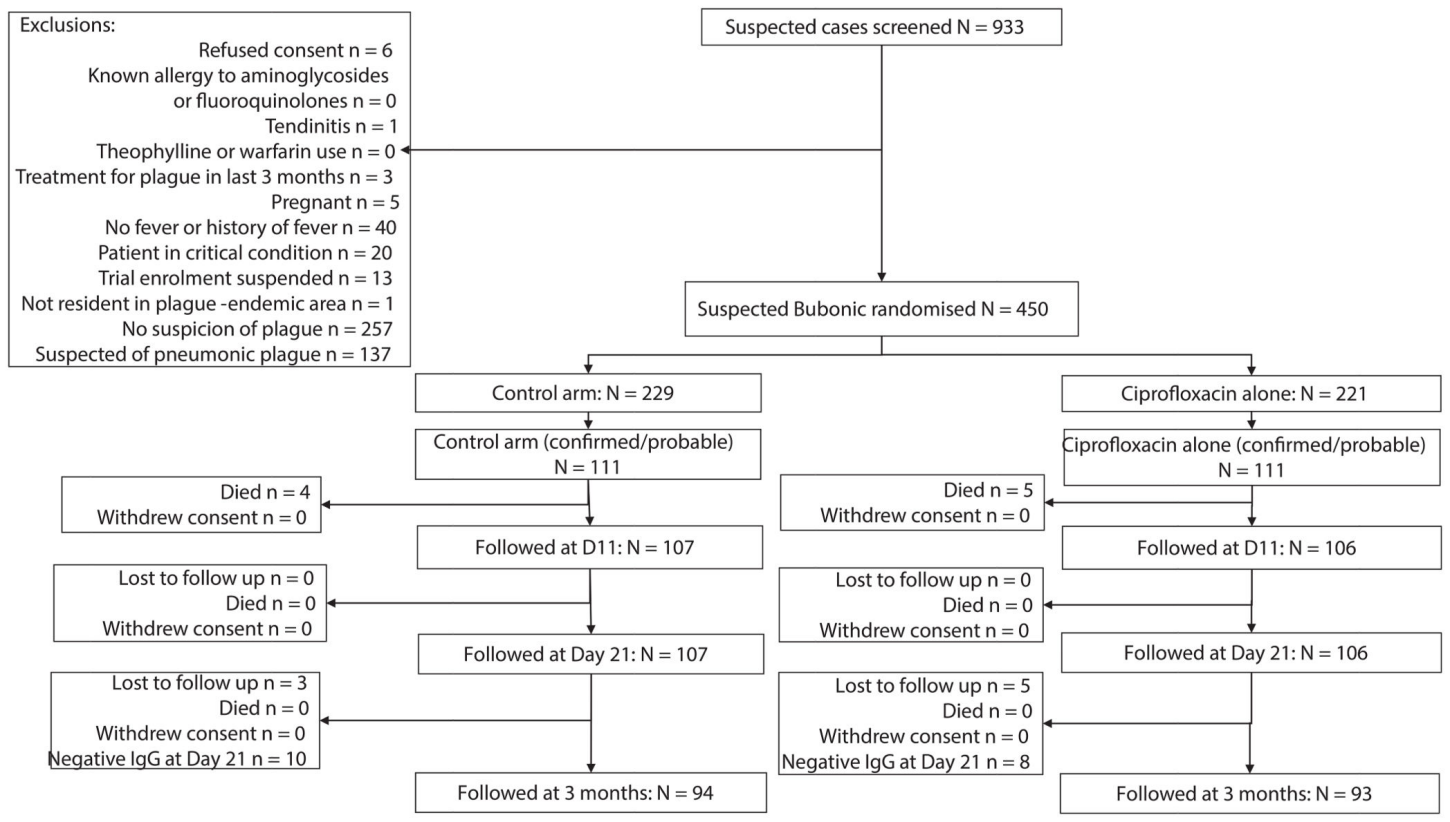
Study Flow Diagram

Recruitment took place at 47 primary and secondary peripheral health centres and hospitals in 12 districts over five transmission seasons during Feb/2020-Mar/2024 (Supplementary Appendix S3 Table). Of 933 suspected bubonic plague patients screened, 450 were enrolled and randomised, with 220 (110 per arm) confirmed and 2 probable infections (Figure 1).

There were 222 patients included in the primary analysis population (ITTI), 449 in the ITT (one patient withdrew consent on Day 6), 444 in the PP, and 221 in the PPI population. Of the 222 in the ITTI population, 220 were laboratory-confirmed (141 (64%) both PCR- and culture-positive; 47 (21%) PCR-positive, culture-negative; 27 (12%) serology-positive only; 5 (2%) culture-positive, PCR-negative), and two probable cases (RDT-positive only), one in each arm (Table 1).

Major deviations for exclusion from the PP analysis population were pre-specified (6/450, 1.3%; control arm ITT: major dosing error (n=1), change to ciprofloxacin (n=2); late follow up (D18 instead of D11, n=1); control arm ITTI: none; intervention arm ITT: withdrawal of consent (n=1), change to ciprofloxacin plus aminoglycoside (n=1); Intervention arm ITTI: change to ciprofloxacin (n=1)). The patient who withdrew consent on day 6 was also excluded from the ITT analysis.

The median age (range) in the ITTI population was 14 (2-72) years and 12 (0-72) years in the ITT (Table 1). The male-to-female ratio was 1.1 and 1.5 in the ITTI and ITT populations, respectively (Table 1). All patients presented with fever and at least one bubo (range for number of buboes per patient 1-5), mostly inguinal (71%) and painful – median pain score 7, range 0-10. Vital signs are in the Supplementary Appendix (S5 Table).

Due to loss of availability, gentamicin replaced streptomycin after the first 20 and 17 patients had been allocated to control and intervention arm, respectively in the ITT population (10 and 9, respectively in the ITTI population, Supplementary Appendix S6 Table). The concurrent randomisation period with gentamicin as the control arm aminoglycoside included 203 confirmed/probable cases; more than the required total sample size of 190 patients for the ITTI analysis.

**Figure 2:**
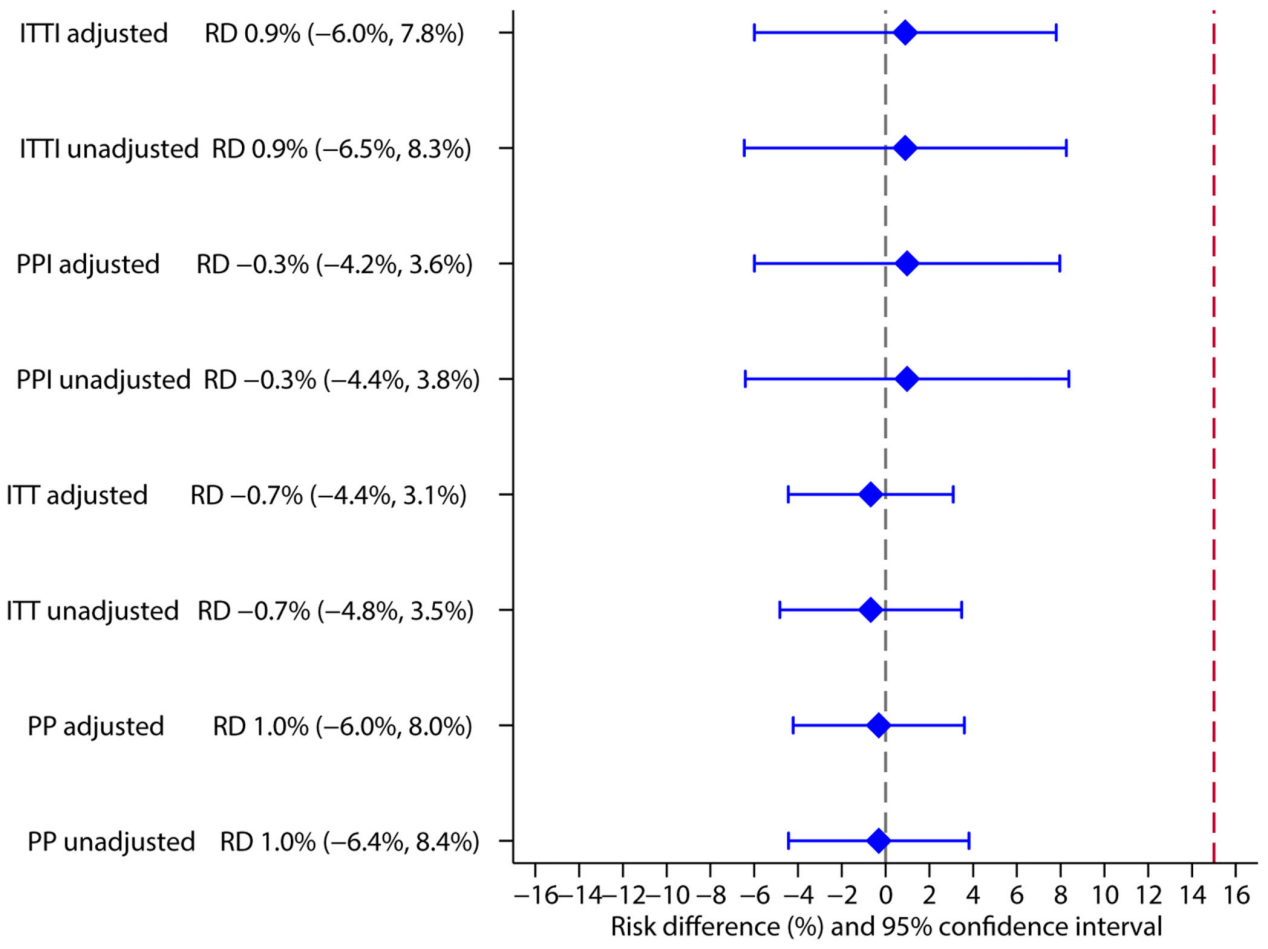
legend: RD: risk difference, ITT: Intention to treat, ITTI: Intention to treat Infected, PP: Per protocol, PPI: Per protocol Infected. Adjusted analyses: adjusted for trial site using robust standard errors in a generalised linear binomial model with an identity link function

In the primary ITTI analysis there were 9 (8.1%) treatment failures in the control and 10 (9.0%) in the intervention arm. The risk difference adjusted for site was 0.9% with an upper confidence bound (UB) of 7.8%, meeting the criterion for non-inferiority (Table 2, Figure 2). The results were similar in the PPI, PP, ITT and unadjusted analyses and for all sensitivity analyses of the ITTI population (Table 2, Supplementary Appendix S7 Table, Supplementary Appendix S8 Figure).

For a secondary outcome that included an additional component of less than 25% reduction in bubo size at D11 as a failure indicator in the composite endpoint, failure rates were 36.0%, (40/111) and 46.8% (51/111) in the control and intervention arm with an adjusted risk difference 10.8% and UB 17.3%. (Supplementary Appendix S9 Table). Amongst patients with measurable buboes at Day 4, approximately one-third had a pain score of <3 (lower pain scores) in each arm (Supplementary Appendix S12 Table) and around half had a pain score of <3 at Day 11.

In both the ITT and ITTI analysis populations, similar percentages of patients in each arm experienced a serious adverse event (SAE), an adverse event (AE) that was serious or not, a non-serious AE and a non-serious adverse drug reaction (Table 3). No SAEs were considered related (investigator assessment) to study drugs.

Overall, 13.8% (62/449) of patients experienced an AE (whether serious or non-serious), 3.6% (16/449) experienced a SAE, and 2.7% (12/449) experienced an adverse drug reaction (Table 3), most commonly, vomiting (3 patients) and diarrhoea (5 patients). The non-serious adverse drug reactions were mild or moderate, except for one severe event of gastrointestinal pain (Supplementary Appendix S10 Table, Supplementary Appendix S11 Table).

## Discussion

IMASOY compared two regimens included in the current international and country’s guidelines and demonstrated that a 10-day course of daily oral ciprofloxacin is non-inferior to a sequential treatment including 3 days injectable aminoglycoside followed by 7 days oral ciprofloxacin. These results are consistent across the intent-to-treat and per-protocol primary analysis populations and sensitivity analyses.

Both treatments were effective, with 90% efficacy and 4% CFR overall. For comparison, during the period of recruitment in the IMASOY-participant districts, the CFR was almost five times as high among bubonic plague patients outside the trial sites (26/138, 19%). Treatment efficacy is not otherwise assessed in routine practice.

IMASOY’s study population is representative of the typical bubonic plague case occurring in Madagascar: during 2020-2024, IMASOY enrolled 220 (61%) of the total 358 total confirmed/probable cases of bubonic plague recorded in the districts where IMASOY recruiting sites were located (unpublished, National Plague Laboratory, hosted by Institut Pasteur Madagascar). The risk of selection bias was reduced by eligibility criteria that excluded only pregnancy (different treatment guidelines) and known prior adverse reactions to study drugs.

The trial was conducted in field conditions, with cases being managed by doctors and nurses as part of their normal practice according to the country’s treatment guidelines, at health facilities ranging from the most peripheral dispensaries to district and regional hospitals. However, patient’s adherence to the prescribed regimen might be higher in the trial than in practice as all cases were hospitalised for the first three days of treatment and were subsequently seen daily by village health workers, which might not be routine practice.

An endpoint of treatment failure at Day 11 that included an additional component for failure of <25% reduction in bubo size was inferior in a comparative analysis, however this composite measure of treatment failure is not considered clinically meaningful, nor objective. Bubo size is inconsistently reported in the literature, with no clear relation to disease severity or resolution.^3^ We also confirmed variable, inaccurate measurement error of buboes by research assistants within and between seasons using a digital calliper in a training exercise using artificial buboes.^21^ Uncertainty and unreliability in the bubo size data is highlighted in the observed differences in analyses for this secondary outcome adjusted for site (inferior) and unadjusted (inconclusive) and the lack of correlation with clinical status; differences are likely due to the variability in measurement error we observed in the artificial bubo measurement exercise between sites and seasons.

Ciprofloxacin was added as first-line treatment in the most recent treatment guidelines of the World Health Organization (WHO) and the United States Centers for Disease Control and Prevention (CDC)^2,14^, and is approved for treating plague by the USA FDA, but based on weak evidence.^22^ IMASOY filled this knowledge gap by generating evidence of the efficacy and safety of two regimens included in Madagascar’s and international treatment guidelines for bubonic plague. However, a regimen including gentamicin – streptomycin is no longer available internationally – has practical limitations as it requires twice-daily injections for three days and is substantially more expensive – approximately 14 US$ for intravenous and 6.5 US$ for intramuscular injections vs. 0.75 US$ for ciprofloxacin alone – plus indirect costs borne by the patient for accommodation and subsistence in several settings in Madagascar. Aminoglycosides also have poor tissue penetration and intracellular activity and are FDA pregnancy category D representing established human foetal risk. Ciprofloxacin has attractive pharmacokinetic properties, including bactericidal activity, good oral bioavailability, good tissue penetration, no need for biochemical monitoring, has an established safety record and is FDA pregnancy category C, whereby benefits may warrant use despite potential risks. Of note, ciprofloxacin proved effective at 500mg twice daily, as per previous WHO guidelines, while the most recent US CDC guidelines have 750mg twice daily. ^14^

There is very little prior knowledge to compare. Mwengee et al ^6^ found no difference between gentamicin and doxycycline in 65 patients in Tanzania, with maximum 19% and 17% failure rates – the corresponding values are 15-16% in each arm in IMASOY. However, direct comparisons are difficult given the different populations, treatments and endpoints.

In conclusion, for the treatment of bubonic plague, ten days of oral ciprofloxacin is an effective alternative to a regimen requiring gentamicin injections.

## Supplementary Material

Supplement

## Figures and Tables

**Table 1:** Baseline characteristics of included participants

		ITT	ITTI	Control	Intervention
N		449	222	111	111
Male (%)		267 (59.5)	118 (53.2)	63 (56.8)	55 (49.5)
Age in years, median (range)		12.0 (0.0 - 72.0)	14.0 (2.0 - 72.0)	14.0 (2.0 - 72.0)	14.0 (2.0 - 64.0)
Receipt of any antibiotic indicated for plague*		31 (6.9)	22 (9.9)	10 (9.0)	12 (10.8)
Receipt of any antibiotic not indicated for plague*		23 (5.1)	12 (5.4)	5 (4.5)	7 (6.3)
Fever		449 (100)	222 (100)	111 (100)	111 (100)
Median duration of fever (range) days		1.0 (0.0 - 8.0)	1.0 (0.0 - 7.0)	1.0 (0.0 - 7.0)	1.0 (0.0 - 7.0)
Seizures		7 (1.6)	6 (2.7)	4 (3.6)	2 (1.8)
Vomiting		41 (9.1)	35 (15.8)	21 (18.9)	14 (12.6)
AVPU†	Alert	431 (95.8)	204 (91.9)	98 (88.3)	106 (95.5)
	Verbal	13 (2.9)	12 (5.4)	9 (8.1)	3 (2.7)
	Painful	6 (1.3)	6 (2.7)	4 (3.6)	2 (1.8)
Number of buboes at baseline, median (range)		1 (1 - 5)	1 (1 - 5)	1 (1 - 5)	1 (1 - 3)
Bubo Location	Armpit	57 (12.7)	41 (18.5)	18 (16.2)	23 (20.7)
	Cervical	69 (15.3)	21 (9.5)	9 (8.1)	12 (10.8)
	Inguinal	321 (71.3)	157 (70.7)	83 (74.8)	74 (66.7)
	Other	0 (0)	0 (0)	0 (0)	0 (0)
Pain score‡, median (range)					
	Armpit	8 (2 - 10)	8 (3 - 10)	8 (4 - 10)	7 (3 - 10)
	Cervical	6.5 (2 - 10)	7.5 (2 - 10)	7 (2 - 10)	8 (4 - 10)
	Inguinal	7 (0 - 10)	7 (0 - 10)	7 (0 - 10)	7 (2 - 10)
	Any location	7 (0 - 10)	7 (0 - 10)	7.5 (0 - 10)	7 (2 - 10)
Diagnosis in the ITTI population (confirmed or probable^§^)	PCR positive, culture positive	-	141	75	66
	PCR positive, culture negative	-	47	19	28
	PCR negative, culture positive	-	5	3	2
	Serological confirmation only	-	27	13	14
	RDT test positive only^§^	-	2	1	1

Data are n (%) unless otherwise stated. ITT: Intention to treat, ITTI: Intention to treat Infected, PCR: Polymerase Chain Reaction

* Not indicated: amoxicillin, penicillin, erythromycin; Indicated: cotrimoxazole, ciprofloxacin, tetracycline, doxycycline, ampicillin

† AVPU: alert, responds to verbal stimulation, responds to pain stimulation only, unresponsive.

‡ In all buboes in each location at baseline.

**Table 2:** Efficacy in the primary and secondary analysis populations

		Primary: Intention to Treat Infected (ITTI)		Per protocol Infected (PPI)	
		Control	Intervention	Control	Intervention
	N	111	111	111	110
Died by Day 11	n (%)	4 (3.6)	5 (4.5)	4 (3.6)	5 (4.5)
Fever at Day 11	n (%)	1 (0.9)	2 (1.8)	1 (0.9)	2 (1.8)
Secondary pneumonic plague	n (%)	3 (2.7)	3 (2.7)	3 (2.7)	3 (2.7)
Extra treatment before or at end of treatment	n (%)	2 (1.8)	2 (1.8)	2 (1.8)	2 (1.8)
Total: treatment failure	n (%, 95% CI)	9 (8.1, 3.8 - 14.8)	10 (9.0, 4.4 - 15.9)	9 (8.1,3.8 - 14.8)	10 (9.1,4.4 - 16.1)	
Unadjusted risk difference (failure)	% (95% CI)	0.9 (-6.5, 8.3)		1.0 (-6.4, 8.4)	
Adjusted risk difference (failure)	% (95% CI)	0.9 (-6.0, 7.8)		1.0 (-6.0, 8.0)	
			
		**Intention to Treat (ITT)**		**Per protocol (PP)**	
	N	229	220	225	219
Died by Day 11	n (%)	4 (1.7)	6 (2.7)	4 (1.8)	6 (2.7)
Fever at Day 11	n (%)	4 (1.7)	2 (0.9)	3 (1.3)	2 (0.9)
Secondary pneumonic plague	n (%)	3 (1.3)	3 (1.4)	3 (1.3)	3 (1.4)
Extra treatment before or at end of treatment	n (%)	3 (1.3)	2 (0.9)	3 (1.3)	2 (0.9)
Total: treatment failure	n (%, 95% CI)	13 (5.7,3.1 - 9.5)	11 (5.0,2.5 - 8.8)	12 (5.3,2.8 - 9.1)	11 (5.0,2.5 - 8.8)
Unadjusted risk difference (failure)	% (95% CI)	-0.7 (-4.8, 3.5)		-0.3 (-4.4, 3.8)	
Adjusted risk difference (failure)	% (95% CI)	-0.7 (-4.4, 3.1)		-0.3 (-4.2, 3.6)	

Adjusted analyses: adjusted for trial site using robust standard errors in a generalised linear binomial model with an identity link function.

**Table 3:** Cumulative safety reporting in the ITT and ITTI population

		ITT population		ITTI population	
		Control	Intervention	Control	Intervention
Randomised	N	229	220	111	111
**Patients with at least one AE (whether serious or not):**					
All	n (%)	35 (15.3)	27 (12.3)	21 (18.9)	20 (18.0)
Adverse drug reaction (ADR)	n (%)	7 (3.1)	5 (2.3)	4 (3.6)	5 (4.5)
Unrelated to study drug	n (%)	29 (12.7)	23 (10.5)	17 (15.3)	16 (14.4)
**Patients with at least one serious adverse event (SAE):**					
All	n (%)	7 (3.1)	9 (4.1)	6 (5.4)	8 (7.2)
Serious adverse drug reaction (SADR)	n (%)	0 (0)	0 (0)	0 (0)	0 (0)
Unrelated to study drug	n (%)	7 (3.1)	9 (4.1)	6 (5.4)	8 (7.2)
**Patients with at least one ADR (whether serious or not) by intensity:**					
Mild (Grade 1)	n (%)	3 (1.3)	3 (1.4)	2 (1.8)	3 (2.7)
Moderate (Grade 2)	n (%)	4 (1.7)	1 (0.5)	2 (1.8)	1 (0.9)
Severe (Grade 3)	n (%)	0 (0)	1 (0.5)	0 (0)	1 (0.9)
Life-threatening (Grade 4)	n (%)	0 (0)	0 (0)	0 (0)	0 (0)
Fatal (Grade 5)	n (%)	0 (0)	0 (0)	0 (0)	0 (0)
**Number of non-serious AE reports received:**					
Total	N	48	33	29	21
Per patient amongst those with an AE	Median (range)	1 (1 - 4)	1 (1 - 3)	1 (1 – 4)	1 (1 – 3)
**Number of SAE reports per patient:**					
Total	N	9	14	8	12
Per patient amongst those with a SAE	Median (range)	1 (1 - 3)	1 (1 - 3)	1 (1 – 3)	1 (1 – 3)
**Patients experiencing SAEs by MedDRA preferred term:**					
Septic shock	N	4	3	4	3
Pneumonic plague	N	3	3	3	3
Sudden death	N	1	1	1	1
Acute respiratory syndrome	N	0	2	0	2
Lymph node rupture	N	0	1	0	1
Seizure	N	1	0	1	0
Vomiting	N	0	1	0	0
Condition aggravated	N	0	1	0	0
Unspecified infection	N	1	0	0	0

ITT: Intention to treat, ITTI: Intention to treat Infected Adverse drug reaction (causality link with the drug: includes improbable, possible and probable as designated by site investigator)
