# Supplementary material for: A trial of ciprofloxacin vs aminoglycoside-ciprofloxacin for bubonic plague
Source: N Engl J Med. Author manuscript; Available in PMC 2025 Sep 9. (PMC7618094; doi:10.1056/NEJMoa2413772)
Supplement: Supplement [file EMS206099-supplement-Supplement.pdf]

Supplementary Appendix

Contents

IMASOY study group 2

IMASOY Oversight Committees 3

Supplemental information about the trial methods 4

Figure S1. Sensitivity analyses: primary efficacy endpoint in the ITTI analysis population 6

Table S1. Treatment regimens 7

Table S2. Analysis populations 8

Table S3. Recruiting districts and age distribution 9

Table S4. Analysis populations 9

Table S5. Vital signs at admission disaggregated by age 10

Table S6. Number of patients randomised preceding and following the protocol amendment allowing the use of any aminoglycoside in the control arm (either streptomycin or gentamicin) 14

Table S7. Sensitivity analyses of primary efficacy outcome (ITTI) 14

Table S8. Secondary efficacy outcome with bubo size reduction component 15

Table S9. Listing of all serious adverse events (SAEs) 16

Table S10. Incidence of all Treatment Emergent Non-serious AE by preferred term and relation (ITT analysis population) 17

Table S11. Incidence of all Treatment Emergent Non-serious AE by preferred term and relation (ITTI analysis population) 18

Table S12. Descriptive secondary outcome summary in ITTI analysis population 19

Table S13 Risk of bias -- GRADE (Cochrane manual): 20

IMASOY study group

CSB II Ivato: Dr RANAIVOSON Tahiry Nadège; CHRR Amoron'I Mania: Dr RAMILISOA Joel, Dr RANDRIA Emmanuel; CSB II Ambalamanakana: ANDRIAMITANTSOA Samison Aurélien; CSB II Ambalalehibe: VITASOA Sahondra Méline; CSB II Kianjandrakefina: RATSIAVICK Zanajefy Saintya; CSB II Ambohimitombo I: SF RAMAHANDRIMANANA Helinirina; CSB II Ambohimitombo II: Dr RALAIVELO Emmerentienne; CSB II Antoetra: RANAIVOSON Santatriniaina; CSB II Mahazina: Dr RANAIVOSON Tahiry Nadège; CSB II Ambatofitorahana: SOANAIHELY Laurence Emilienne; CSB II Volafotsy: Dr RAKOTONIRAINY Chantal; CSB II Ambohipo Sud: Dr KIRIKOU Marie Laurencia; CHRD/ CSB II Ambovombe Centre: Dr RASOAMANANJARANDRAINY Eliane; CSB II Anjoman'Akona: RANOROVAOARILALA Philipien; CSBII Anjoma Nandihizana: ANJARASOA Sandra Mamy Beatrice, RAHANITRINIAINA Vohanginirina Marie; CSB II Talata Vohimena: Dr RANDRIANAMPARANY Hajanirina Romi; CSB II Ambohimilanja: HANITRINIAINA Marie Claire; CSB II Ambohimahazo: RAFANOMEZANTSOA Jeannine Pierrett; CSB II Mahatoka: RAFANOMEZANTSOA Mickaël Ben Issa; CSB II Sahamandresy: TOJONIAINA Jery Rémi; CSB II Asabotsy Anjiro: Dr RAKOTOHARIMANANA Hariniony; CSB II Andapa: ANITRINIAINA Oly Zinah; CSB II Morarano: Dr RAKOTOMALALA Narisoa; CSB I Mangarivotra: RAHOLINJATOVO Prisca Anick; CHRR Bongolava: RANDRIANTSIZAFY Fenosoa: CSB II Ambalanirana: Dr TATA Helmogène; CSBII Ankadin'ondry Sakay: Dr RAMAROKOTO Nirinjato Harivelo; CSB II Andraramila: RASOLOHERY Raymond Chrysanthe; CSBII Andranomiditra: RANDRIANIRINA Aimé Frédéric; CSB II Fiadanana: Dr RAKOTOMAVO Voriasy Sandere; CSB II Camp Robin: Dr RANDRIANIRINA Gaston; CHRD Ambohimahasoa: Dr RAKOTOVOAVY Voharilalaina Mamy; CSB II Sahatona: Dr TOMBOARIMALALANIRINA Lea Haingotiana Charline; CSBII Ambohinamboarina: RAKOTONIRINA Marcel; CHRD Ankazobe: Dr RANDRIANALIMALALA Jean Dénis, Dr RAKOTONDRINA Eugène; CSB II Miantso: Dr RABIAKELY Vohomby Aristide; CSBII Ambohitromby: Dr RAZAKARISOA Gregoire Joseph; CHRD Anjozorobe: Dr Lova Andriamanantsoa, Dr Andriamanlinarivo Vololonirina; CSB II Analaroa: RASOAMBOLANORO Felanarisoa Ravakaniaina; CSBII Ambohimirary: HASIMBOLANIRINA Larissa; CSBII Ambogamarina: RAZOELIMANANA Marosoa; CSB II Ambatolaona:

RABEARILALA Jean Marie; CSBII Ambohidratrimoanala: Dr RAHARINJATO Fitia; CSBII Saoavina: Dr RAZAIARIMANANA Faramalala; CSB II Andolofotsy: Dr RAZAKA Josette; CSB II Miandrandra: RAHANITRINIAINA Léonore Nina; Division de lutte contre la peste: Dr RANDRIAMANANTSOA Mamy Gabriel; Medecin inspecteur Ambositra: Dr RAZAFINDRATSIMABOZAKA Nestor; Medecin inspecteur Miarinarivo: RAKOTONDRAMARO Tina Germain; Medecin inspecteur Manandriana: Dr RANDRIAMAMPIONONA Harivelo; Medecin inspecteur Ambohimahasoa: Dr RAKOTOMAHENINA N. Jocelyne; Medecin inspecteur Vohibato: Dr RAKOTONDRAMANANA Fréderic; Medecin inspecteur Moramanga: Dr RAZANAJATOVO Hery Nomenjanahary; Medecin inspecteur Mananjakandriana: Dr ANDRIAMAHAZO Marie Josée; Medecin inspecteur Anjozorobe: Dr ANDRIAMANANTSOA Veloharinosy Lovalalaina, Dr RAZAFINDRAKOTONARISON Serge André; Médecin inspecteur Tsiroanomandidy: Dr RALAMBOZAFY Maro Hosea, Dr ANDRIAMANANTSOA Dinanirina; Médecin inspecteur Betafo: Dr ANDRIAMITANTSOA Fidy Nirina; Medecin inspecteur Ankazobe: Dr ANDRIAMANJATO Jean David; Medecin inspecteur Arivonimamo: Dr ANDRIAMISANDRATRA Maherisoa

IMASOY Oversight Committees

IMASOY Trial Operations Group: Mihaja Raberahona (Chair); Piero Olliaro, Rindra Vatosoa Randremanana, Josephine Bourner, Ravaka Randriamparany; Tsinjo Rasoanaivo; Lisy Hanitra Razananaivo; Gabriella Zadonirina; Théodora Mayouya-Gamana; Reziky Tiandraza Mangahasimbola; Elise Pesonel; Tansy Edwards

IMASOY Trial Steering Committee: Alexandra Calmy (Chair); Peter Horby; Mihaja Raberahona; Piero Olliaro; Rindra Vatosoa Randremanana; Tansy Edwards

IMASOY Data Safety Monitoring Committee: David Lalloo (Chair); Paul Mead; Julia Simpson

IMASOY Writing Committee: Peter Horby; Mihaja Raberahona; Piero Olliaro; Rindra Vatosoa Randremanana; Tansy Edwards, Josephine Bourner, Minoarisoa Rajerison

Supplemental information about the trial methods

Study cohorts: In addition to the randomised cohort of bubonic plague cases described in the main paper, the trial also has an embedded pneumonic plague observational cohort (n=13) and social science sub-study investigating the acceptability of a ciprofloxacin monotherapy among local clinicians, which will be reported separately.

Amendments: Three important changes to the trial design have occurred since the start of recruitment in February 2020.

First, the trial was initiated with streptomycin + ciprofloxacin as the control arm treatment. However, due to the expiration of streptomycin stock across Madagascar in January 2021, which was not locally renewed due to global shortages, patients who were randomised to the control arm received the new first-line standard of care treatment for bubonic plague, gentamicin + ciprofloxacin. The protocol was therefore amended to account for the evaluation of an aminoglycoside (streptomycin or gentamicin) + ciprofloxacin in the control group.

Second, until August 2021 patients with either suspected bubonic or pneumonic plague were randomised to trial treatment. However, following the release of updated guidance from the United States Centers for Disease Control and Prevention recommending combination therapy for the treatment of pneumonic plague, the IMASOY protocol was amended and patients with suspected pneumonic plague were no longer randomised to a monotherapy. Instead, these patients were enrolled into an observational cohort in which standard of care was given by the patient's treating clinician according to national treatment guidelines. Data continued to be collected from this cohort with the aim of characterising disease evolution and patient outcomes. Patients with suspected pneumonic plague were eligible to participate in the observational study if they had a recent onset of fever, cough, tachypnoea, and an epidemiological link with a confirmed or probable case of primary or secondary pneumonic plague within 7 days of symptom onset.

Finally, the primary endpoint was amended to remove a component of the composite endpoint that included a 25% decrease in bubo size.

This decision was made following the publication of a study evaluating the measurement error for buboes of different shapes and sizes, when using a digital calliper on artificial buboes specifically created to mimic patient buboes for trial field training.[1] Consistent and accurate measurement of buboes, that may be of varying shapes and volumes, in field conditions without specialist imaging equipment is challenging. The study found substantial variability in measurement error and limited precision, particularly for smaller buboes and there was significant variation in measurement error between sites and seasons. The results of this study raised serious concern about the impact of measurement error and subjectivity on the assessment of the composite primary endpoint of the IMASOY trial, particularly for smaller buboes, thus justifying the amendment to the primary endpoint to a more objective set of composite outcome components. The original composite endpoint was retained as a new secondary endpoint for interest.

Further, it should be noted that bubo size and evolution was of clinical descriptive interest but is not a meaningful clinical indication of treatment response and bubo evolution does not dictate patient management.

Figure S1. Sensitivity analyses: primary efficacy endpoint in the ITTI analysis population

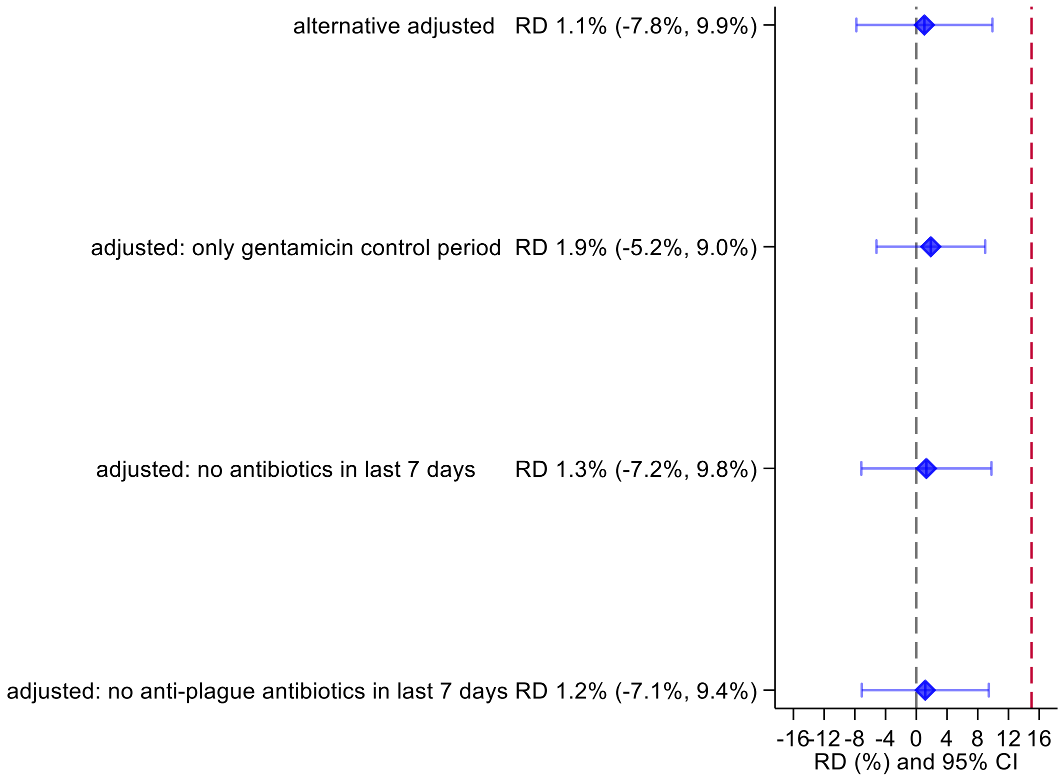

Legend: RD: risk difference, CI: confidence interval. Alternative adjusted: fixed effects for age and sex and a robust standard error adjustment for season rather than site. Other adjusted sub-group analyses shown here include a robust standard error adjustment for site.

Table S1. Treatment regimens

ADULTS AND CHILDREN ≥15 YEARS<br>First-line treatment for bubonic plague										
Day	1	2	3	4	5	6	7	8	9	10
Adult	STREPTOMYCIN 1g IM every 12 hours			CIPROFLOXACIN 500mg PO every 12 hours						
Children ≥15 years	STREPTOMYCIN 15mg/kg IM (max. 1g) every 12 hours			CIPROFLOXACIN 15mg/kg PO (max. 500mg) every 12 hours						

ADULTS AND CHILDREN ≥15 YEARS<br>Alternative treatment regimen for bubonic plague when streptomycin is unavailable<br>Gentamicin is preferentially given by the IV route										
Day	1	2	3	4	5	6	7	8	9	10
Adult	GENTAMICIN 2.5mg/kg IV or IM every 12 hours			CIPROFLOXACIN 500mg PO every 12 hours						
Children ≥15 years	GENTAMICIN 2.5mg/kg IV or IM every 12 hours			CIPROFLOXACIN 15mg/kg PO (max. 500mg) every 12 hours						

CHILDREN <15 YEARS<br>Alternative treatment regimen if it is not possible to give the oral route initially										
Day	1	2	3	4	5	6	7	8	9	10
First-line	CIPROFLOXACIN 15mg/kg PO (max. 500mg) every 12 hours									
Alternative	STREPTOMYCIN 15mg/kg IM (max. 1g) every 12 hours			CIPROFLOXACIN 15mg/kg PO (max. 500mg) every 12 hours						

IN THE EVENT OF AN ABSOLUTE CONTRAINDICATION AGAINST AMINOGLYCOSIDES<br>Ciprofloxacin may be taken orally as soon as the patient's clinical condition allows, if treatment has been started by the IV route.										
Day	1	2	3	4	5	6	7	8	9	10
First-line	CIPROFLOXACIN 15mg/kg PO (max. 500mg) every 12 hours									
Alternative	CIPROFLOXACIN 15mg/kg PO (max. 500mg) every 12 hours or IV (max. 400mg) every 12 hours									

PREGNANT WOMEN<br>Alternative treatment regimen if it is not possible to give the oral route initially<br>Gentamicin is preferentially given by the IV route										
Day	1	2	3	4	5	6	7	8	9	10
First-line	CIPROFLOXACIN 500mg every 12 hours									
Alternative	GENTAMICIN 2.5mg/kg IV or IM every 12 hours			CIPROFLOXACIN 500mg every 12 hours						

Analysis populations: Four analysis populations were defined in the trial (**Table S2**). The Intention to Treat Infected (ITTI) for bubonic plague cases was the primary analysis population. The analysis was repeated in the bubonic plague cohort in the intention to treat (ITT), per protocol (PP) and per protocol infected (PPI) cohorts.

The safety analysis population was conducted in patients who received at least one dose of treatment.

Table S2. Analysis populations

	**Intention to treat (ITT)**	**Intention to treat infected (ITTI)**	**Per protocol (PP)**	**Per protocol infected (PPI)**
Bubonic plague (all cases)	X		X	
Bubonic plague (confirmed and probable cases)		X		X

Sensitivity analyses: Pre-specified sensitivity analyses include ITTI primary efficacy non-inferiority comparisons adjusted for site in i) the concurrent randomisation period for which gentamicin was the aminoglycoside given in the control arm, ii) patients who did not receive any antibiotic treatment in the seven days prior to enrolment, iii) patient patients who did not receive anti-plague antibiotic treatments in the seven days prior to enrolment and iv) an alternative adjusted analysis with fixed effects for age and sex and a robust standard error adjustment for season rather than site. Antibiotic treatment considered indicated for plague (anti-plague) could be any of the following, cotrimoxazole, ciprofloxacin, tetracycline, doxycycline, ampicillin.

Table S3. Recruiting districts and age distribution

		Randomised (ITT)	ITT Infected	Control	Intervention
N		449	222	111	111
District	Ambohimahasoa	144 (32.1)	70 (31.5)	32 (28.8)	38 (34.2)
	Ambositra	124 (27.6)	76 (34.2)	42 (37.8)	34 (30.6)
	Anjozorobe	22 (4.9)	8 (3.6)	4 (3.6)	4 (3.6)
	Ankazobe	12 (2.7)	10 (4.5)	3 (2.7)	7 (6.3)
	Arivonimano	0 (0)	0 (0)	0 (0)	0 (0)
	Betafo	4 (0.9)	3 (1.4)	2 (1.8)	1 (0.9)
	Manandriana	110 (24.5)	48 (21.6)	22 (19.8)	26 (23.4)
	Manjakandriana	4 (0.9)	4 (1.8)	3 (2.7)	1 (0.9)
	Miarinarivo	2 (0.4)	1 (0.5)	1 (0.9)	0 (0)
	Moramanga	8 (1.8)	2 (0.9)	2 (1.8)	0 (0)
	Tsiroanomandidy	6 (1.3)	0 (0)	0 (0)	0 (0)
	Vohibato	13 (2.9)	0 (0)	0 (0)	0 (0)
Age (years)	<1	1 (0.2)	0 (0)	0 (0)	0 (0)
	1-4	67 (14.7)	17 (7.7)	8 (7.2)	9 (8.1)
	5-9	96 (21.4)	35 (15.8)	14 (12.6)	21 (18.9)
	10-14	123 (27.4)	69 (31.1)	37 (33.3)	32 (28.8)
	15-19	49 (10.9)	27 (12.2)	16 (14.4)	11 (9.9)
	20-39	83 (18.5)	48 (21.6)	20 (18.0)	28 (25.2)
	40+	31 (6.9)	26 (11.7)	16 (14.4)	10 (9.0)

ITT: intention-to-treat. One patient withdrew consent on Day 6 amongst the suspected cases.

Table S4. Analysis populations

Population	Total	Control	Intervention
Intention-to-treat all: ITT	449	229	220
Intention-to-treat infected: ITTI	222	111	111
Per protocol all: PP	444	225	219
Per protocol infected: PPI	221	111	110

Major deviations excluded from per-protocol analysis population:
Control arm ITT: major dosing error (n=1), change to ciprofloxacin (n=2); late follow up (D18 instead of D11, n=1)
Control arm ITTI: none
Intervention arm ITT: withdrawal of consent (n=1), change to ciprofloxacin plus aminoglycoside (n=1)
Intervention arm ITTI: change to ciprofloxacin (n=1)
The patient who withdrew consent on day 6 is also excluded from the ITT analysis.

Table S5. Vital signs at admission disaggregated by age

		Randomised (ITT)	ITT Infected	Control	Monotherapy
**<5 years old:**					
	N	67	17	8	9
**Respiratory rate (cycles/min)**	Measurements, n	66	17	8	9
	Mean (SD)	28.5 (6.2)	31.3 (7.2)	30.4 (8.0)	32.1 (6.7)
	Median (range)	28.0 (16.0, 45.0)	30.0 (18.0, 45.0)	29.5 (18.0, 45.0)	30.0 (22.0, 41.0)
**Heart rate (beats/min)**	Measurements, n	66	17	8	9
	Mean (SD)	106.6 (19.8)	111.4 (27.6)	114.8 (33.0)	108.3 (23.4)
	Median (range)	102.0 (73.0, 162.0)	102.0 (74.0, 162.0)	102.5 (78.0, 162.0)	102.0 (74.0, 142.0)
**Systolic BP (mmHg)**	Measurements, n	63	16	7	9
	Mean (SD)	94.7 (10.9)	96.3 (15.4)	97.3 (12.4)	95.6 (18.1)
	Median (range)	90.0 (70.0, 140.0)	90.0 (80.0, 140.0)	91.0 (80.0, 110.0)	90.0 (80.0, 140.0)
**Diastolic BP (mmHg)**	Measurements, n	63	16	7	9
	Mean (SD)	59.4 (10.7)	61.3 (12.5)	64.3 (15.0)	58.9 (10.5)
	Median (range)	60.0 (40.0, 89.0)	60.0 (40.0, 87.0)	60.0 (50.0, 87.0)	60.0 (40.0, 80.0)
**Axillary temperature (°C)**	Measurements, n	67	17	8	9
	Mean (SD)	38.9 (0.9)	39.4 (0.9)	39.6 (0.7)	39.1 (1.0)
	Median (range)	39.0 (37.2, 41.5)	39.6 (37.8, 40.3)	39.9 (38.5, 40.2)	39.4 (37.8, 40.3)
**Oxygen saturation (%)**	Measurements, n	63	16	7	9
	Mean (SD)	96.0 (2.6)	96.4 (2.1)	95.7 (2.2)	96.9 (1.9)
	Median (range)	97.0 (87.0, 99.0)	96.0 (92.0, 99.0)	96.0 (92.0, 98.0)	96.0 (94.0, 99.0)
**5-9 years old:**					
	N	96	35	14	21
**Respiratory rate (cycles/min)**	Measurements, n	96	35	14	21
	Mean (SD)	26.4 (5.1)	26.2 (5.9)	26.2 (3.9)	26.1 (7.0)

		Randomised (ITT)	ITT Infected	Control	Monotherapy
	Median (range)	25.5 (16.0, 48.0)	25.0 (16.0, 48.0)	27.0 (21.0, 32.0)	25.0 (16.0, 48.0)
**Heart rate (beats/min)**	Measurements, n	96	35	14	21
	Mean (SD)	104.9 (18.0)	107.7 (21.9)	109.2 (18.4)	106.6 (24.4)
	Median (range)	103.0 (66.0, 161.0)	103.0 (66.0, 161.0)	110.0 (75.0, 146.0)	100.0 (66.0, 161.0)
**Systolic BP (mmHg)**	Measurements, n	95	35	14	21
	Mean (SD)	95.1 (9.7)	96.4 (10.6)	95.7 (10.9)	96.8 (10.7)
	Median (range)	95.0 (70.0, 120.0)	100.0 (70.0, 120.0)	100.0 (70.0, 110.0)	100.0 (80.0, 120.0)
**Diastolic BP (mmHg)**	Measurements, n	95	35	14	21
	Mean (SD)	58.6 (10.2)	61.2 (11.8)	59.3 (12.1)	62.5 (11.7)
	Median (range)	60.0 (40.0, 90.0)	60.0 (40.0, 90.0)	60.0 (40.0, 90.0)	60.0 (40.0, 80.0)
**Axillary temperature (°C)**	Measurements, n	96	35	14	21
	Mean (SD)	38.8 (0.9)	39.3 (0.8)	39.1 (0.9)	39.4 (0.8)
	Median (range)	38.7 (36.5, 41.2)	39.1 (37.7, 41.2)	38.9 (37.7, 41.0)	39.2 (38.0, 41.2)
**Oxygen saturation (%)**	Measurements, n	96	35	14	21
	Mean (SD)	96.1 (2.1)	96.2 (2.1)	96.6 (1.3)	96.0 (2.5)
	Median (range)	96.0 (89.0, 99.0)	97.0 (89.0, 99.0)	96.5 (94.0, 99.0)	97.0 (89.0, 99.0)
**10-17 years old:**					
	N	154	86	45	41
**Respiratory rate (cycles/min)**	Measurements, n	153	85	45	40
	Mean (SD)	23.1 (4.5)	23.0 (4.8)	23.7 (5.3)	22.3 (4.1)
	Median (range)	23.0 (14.0, 42.0)	23.0 (14.0, 36.0)	23.0 (14.0, 36.0)	23.5 (14.0, 28.0)
**Heart rate (beats/min)**	Measurements, n	152	84	45	39
	Mean (SD)	93.7 (14.7)	96.6 (15.5)	97.5 (15.2)	95.7 (16.0)
	Median (range)	93.5 (59.0, 134.0)	98.0 (65.0, 134.0)	98.0 (65.0, 134.0)	98.0 (65.0, 133.0)
**Systolic BP (mmHg)**	Measurements, n	152	84	45	39

		Randomised (ITT)	ITT Infected	Control	Monotherapy
	Mean (SD)	100.2 (11.8)	100.2 (12.7)	99.4 (12.4)	101.2 (13.1)
	Median (range)	100.0 (70.0, 130.0)	100.0 (70.0, 130.0)	100.0 (72.0, 120.0)	100.0 (70.0, 130.0)
**Diastolic BP (mmHg)**	Measurements, n	152	84	45	39
	Mean (SD)	61.0 (10.4)	62.3 (11.5)	62.8 (11.0)	61.6 (12.1)
	Median (range)	60.0 (40.0, 96.0)	60.0 (40.0, 96.0)	60.0 (40.0, 96.0)	60.0 (40.0, 90.0)
**Axillary temperature (°C)**	Measurements, n	154	86	45	41
	Mean (SD)	38.8 (1.0)	39.2 (0.9)	39.4 (0.9)	39.1 (1.0)
	Median (range)	38.7 (36.6, 41.2)	39.3 (37.5, 41.2)	39.5 (37.6, 41.1)	39.0 (37.5, 41.2)
**Oxygen saturation (%)**	Measurements, n	150	82	44	38
	Mean (SD)	96.1 (2.6)	95.6 (2.9)	95.5 (3.2)	95.8 (2.6)
	Median (range)	97.0 (84.0, 99.0)	96.5 (84.0, 99.0)	96.0 (84.0, 99.0)	97.0 (90.0, 99.0)
**18+ years old:**					
	N	132	84	44	40
**Respiratory rate (cycles/min)**	Measurements, n	128	81	42	39
	Mean (SD)	21.0 (4.2)	21.5 (4.8)	21.1 (4.8)	21.9 (4.8)
	Median (range)	20.0 (14.0, 42.0)	21.0 (14.0, 42.0)	20.0 (14.0, 35.0)	21.0 (17.0, 42.0)
**Heart rate (beats/min)**	Measurements, n	128	81	42	39
	Mean (SD)	82.1 (14.1)	84.8 (14.8)	86.2 (16.0)	83.4 (13.5)
	Median (range)	81.0 (45.0, 130.0)	86.0 (60.0, 130.0)	81.0 (60.0, 130.0)	87.0 (60.0, 102.0)
**Systolic BP (mmHg)**	Measurements, n	130	83	43	40
	Mean (SD)	108.6 (14.3)	106.2 (14.2)	107.3 (14.9)	105.1 (13.4)
	Median (range)	110.0 (70.0, 160.0)	100.0 (70.0, 160.0)	110.0 (70.0, 160.0)	100.0 (70.0, 140.0)
**Diastolic BP (mmHg)**	Measurements, n	130	83	43	40
	Mean (SD)	66.2 (11.2)	65.2 (11.1)	67.5 (11.8)	62.8 (9.9)
	Median (range)	62.5 (40.0, 99.0)	60.0 (40.0, 90.0)	62.0 (45.0, 90.0)	60.0 (40.0, 90.0)

		Randomised (ITT)	ITT Infected	Control	Monotherapy
**Axillary temperature (oC)**	Measurements, n	132	84	44	40
	Mean (SD)	38.6 (0.9)	38.9 (0.9)	39.0 (0.9)	38.8 (0.9)
	Median (range)	38.5 (35.3, 41.4)	39.0 (37.4, 41.4)	39.0 (37.4, 41.4)	39.0 (37.5, 40.3)
**Oxygen saturation (%)**	Measurements, n	128	81	42	39
	Mean (SD)	96.0 (2.3)	96.0 (2.2)	96.2 (1.6)	95.8 (2.6)
	Median (range)	96.5 (87.0, 99.0)	96.0 (88.0, 99.0)	96.0 (92.0, 99.0)	96.0 (88.0, 99.0)

Table S6. Number of patients randomised preceding and following the protocol amendment allowing the use of any aminoglycoside in the control arm (either streptomycin or gentamicin)

Recruitment period	Streptomycin & ciprofloxacin	Gentamicin & ciprofloxacin	Ciprofloxacin
All patients (ITT)*:			
On or before 03 Feb 2021	20	0	17
On or after 12 Feb 2021	0	209	204
Confirmed/probable patients (ITTI):			
On or before 03 Feb 2021	10	0	9
On or after 12 Feb 2021	0	101	102

ITT: intention to treat, ITTI: intention to treat infected

* Including the patient who withdrew after day 6

Table S7. Sensitivity analyses of primary efficacy outcome (ITTI)

	Control	Ciprofloxacin
Sensitivity: ITTI primary (adjusted for sex, age as fixed effects and clustering by season)	111	111
Total: composite failure or death, n (%)	9 (8.1)	10 (9.0)
Adjusted risk difference % (one-sided 2.5%CI UB)		1.1 (-7.8, 9.9)
Sensitivity: ITTI primary (excluding the period with streptomycin in control arm, adjusted for site)	101	102
Total: composite failure or death, n (%)	7 (6.9)	9 (8.8)
Adjusted risk difference % (95%CI UB)		1.9 (-5.2, 9.0)
Sensitivity: ITTI primary (excluding those with antibiotic treatment in last 7 days, adjusted for site)	98	95
Total: composite failure or death, n (%)	8 (8.2)	9 (9.5)
Adjusted risk difference % (95%CI UB)		1.3 (-7.2, 9.8)
Sensitivity: ITTI primary (excluding those with anti-plague antibiotic treatment in last 7 days, adjusted for site)	101	99
Total: composite failure or death, n (%)	8 (7.9)	9 (9.1)
Adjusted risk difference % (95%CI UB)		1.2 (-7.1, 9.4)

Failure of intervention vs control: positive difference values correspond to more failure in intervention arm and therefore need to see an upper bound (UB) < 15% for non-inferiority. Aminoglycoside period and no previous antibiotic analyses adjusted for possible clustering by site using robust standard errors. RD=risk difference, CI=confidence interval, ITTI = intention-to-treat infected. Site adjustments using robust standard errors.

Table S8. Secondary efficacy outcome with bubo size reduction component

		Control	Ciprofloxacin
Secondary ITTI analysis	N	111	111
Died by D11	n (%)	4 (3.6)	5 (4.5)
Fever at D11	n (%)	1 (0.9)	2 (1.8)
Secondary pneumonic plague	n (%)	3 (2.7)	3 (2.7)
Extra treatment before or at end of treatment	n (%)	2 (1.8)	2 (1.8)
Less than 25% reduction in bubo size	n (%)	35 (31.5)	46 (41.4)
Total: treatment failure		40 (36.0, 3.8 - 14.8)	52 (46.8, 4.4 - 15.9)
Unadjusted risk difference (failure)	% (95% CI)		10.8 (-2.1, 23.7)
Adjusted risk difference (failure)	% (95% CI)		10.8 (4.3, 17.3)

Table S9. Listing of all serious adverse events (SAEs)

Arm	Patient	SAE number	MedDRA preferred term	Intensity	Relation	Onset (day)	Died (day)
**Confirmed case (ITTI)**							
ciprofloxacin alone	1	1	Sudden death	Fatal	None	2	2
control	2	1	Sudden death	Fatal	None	2	2
ciprofloxacin alone	3	1	Pneumonic plague	Life threatening	None	1	
		2	Acute respiratory distress syndrome	Life threatening	None	1	
		3	Septic shock	Fatal	None	1	1
ciprofloxacin alone	4	1	Pneumonic plague	Severe	None	4	
control	5	1	Pneumonic plague	Life threatening	None	3	
control	6	1	Septic shock	Fatal	None	2	2
ciprofloxacin alone	7	1	Lymph node rupture	Severe	None	11	
control	8	1	Pneumonic plague	Life threatening	None	2	
ciprofloxacin alone	9	1	Lymph node rupture	Severe	None	21	
control	10	1	Pneumonic plague	Life threatening	None	2	
		2	Seizure	Severe	None	2	
		3	Septic Shock	Fatal	None	2	2
ciprofloxacin alone	11	1	Septic shock	Fatal	None	1	1
ciprofloxacin alone	12	1	Septic shock	Fatal	None	1	1
ciprofloxacin alone	13	1	Pneumonic plague	Life threatening	None	3	
		2	Acute respiratory distress syndrome	Life threatening	None	3	
		3	Septic shock	Fatal	None	3	3
control	14	1	Septic shock	Fatal	None	1	1
**Suspected case (ITT)**							
ciprofloxacin alone	15	1	Vomiting	Severe	None	4	
		2	Condition aggravated	Fatal	None	8	8
control	16	1	Unspecified infection	Moderate	None	8	

Table S10. Incidence of all Treatment Emergent Non-serious AE by preferred term and relation (ITT analysis population)

		Control N = 229		Intervention N = 220		All N = 449	
System Organ Class	Preferred Term	Not related	Related	Not related	Related	Not related	Related
Ear and labyrinth disorders	Hypoacusis	0	1 (0.4)	0	0	0	1 (0.2)
	Vertigo	0	1 (0.4)	1 (0.5)	0	1 (0.2)	1 (0.2)
Gastrointestinal disorders	Abdominal distension	0	0	1 (0.5)	0	1 (0.2)	0
	Abdominal pain	0	0	1 (0.5)	0	1 (0.2)	0
	Abdominal pain upper	0	0	1 (0.5)	0	1 (0.2)	0
	Diarrhoea	3 (1.3)	3 (1.3)	2 (0.9)	2 (0.9)	5 (1.1)	5 (1.1)
	Gastrointestinal pain	0	0	0	1 (0.5)	0	1 (0.2)
	Vomiting	7 (3.1)	1 (0.4)	5 (2.3)	2 (0.9)	12 (2.7)	3 (0.7)
General disorders and administration site conditions	Asthenia	1 (0.4)	0	4 (1.8)	0	5 (1.1)	0
	Chills	0	0	1 (0.5)	0	1 (0.2)	0
	Hyperthermia	1 (0.4)	0	2 (0.9)	0	3 (0.7)	0
	Pyrexia	6 (2.6)	0	3 (1.4)	0	9 (2.0)	0
Injury, poisoning and procedural complications	Fall	0	0	2 (0.9)	0	2 (0.4)	0
	Wound	2 (0.9)	0	0	0	2 (0.4)	0
Metabolism and nutrition disorders	Dehydration	0	0	1 (0.5)	0	1 (0.2)	0
Musculoskeletal and connective tissue disorders	Flank pain	0	1 (0.4)	0	0	0	1 (0.2)
	Myalgia	0	1 (0.4)	0	0	0	1 (0.2)
Nervous system disorders	Headache	4 (1.7)	1 (0.4)	1 (0.5)	0	5 (1.1)	1 (0.2)
	Seizure	3 (1.3)	0	0	0	3 (0.7)	0
Respiratory, thoracic and mediastinal disorders	Chest pain	2 (0.9)	0	0	0	2 (0.4)	0
	Cough	6 (2.6)	0	0	1 (0.5)	6 (1.3)	1 (0.2)
	Dyspnoea	2 (0.9)	0	0	0	2 (0.4)	0
	Haemoptysis	1 (0.4)	0	0	0	1 (0.2)	0
Skin and subcutaneous tissue disorders	Perioral dermatitis	1 (0.4)	0	0	0	1 (0.2)	0
	Pruritus	0	0	1 (0.5)	0	1 (0.2)	0
Vascular disorders	Hypotension	0	0	1 (0.5)	0	1 (0.2)	0

Data are n (%) of patients randomised. NR = Not related, ADR = Adverse Drug Reaction

Table S11. Incidence of all Treatment Emergent Non-serious AE by preferred term and relation (ITTI analysis population)

		Control N = 111		Intervention N = 111		All N = 222	
System Organ Class	Preferred Term	Not related	Related	Not related	Related	Not related	Related
Ear and labyrinth disorders	Hypoacusis	0	1 (0.9)	0	0	0	1 (0.5)
Gastrointestinal disorders	Abdominal pain	0	0	1 (0.9)	0	1 (0.5)	0
	Diarrhoea	2 (1.8)	3 (2.7)	2 (1.8)	2 (1.8)	4 (1.8)	5 (2.3)
	Gastrointestinal pain	0	0	0	1 (0.9)	0	1 (0.5)
	Vomiting	6 (5.4)	0	2 (1.8)	2 (1.8)	8 (3.6)	2 (0.9)
General disorders and administration site conditions	Asthenia	0	0	3 (2.7)	0	3 (1.4)	0
	Hyperthermia	1 (0.9)	0	2 (1.8)	0	3 (1.4)	0
	Pyrexia	2 (1.8)	0	3 (2.7)	0	5 (2.3)	0
Injury, poisoning and procedural complications	Wound	1 (0.9)	0	0	0	1 (0.5)	0
Metabolism and nutrition disorders	Dehydration	0	0	1 (0.9)	0	1 (0.5)	0
Nervous system disorders	Headache	3 (2.7)	0	0	0	3 (1.4)	0
	Seizure	3 (2.7)	0	0	0	3 (1.4)	0
Respiratory, thoracic and mediastinal disorders	Chest pain	1 (0.9)	0	0	0	1 (0.5)	0
	Cough	3 (2.7)	0	0	1 (0.9)	3 (1.4)	1 (0.5)
	Dyspnoea	2 (1.8)	0	0	0	2 (0.9)	0
	Haemoptysis	1 (0.9)	0	0	0	1 (0.5)	0
Vascular disorders	Hypotension	0	0	1 (0.9)	0	1 (0.5)	0

Data are n (%) of patients randomised

NR = Not related, ADR = Adverse Drug Reaction

Table S12. Descriptive secondary outcome summary in ITTI analysis population

		Control		Intervention	
		N		N	
Fever at D4	n (%)	107	4 (3.7)	106	5 (4.7)
Developed secondary pneumonic plague	n (%)	111	3 (2.7)	111	3 (2.7)
Experienced a SAE by D4	n (%)	111	6 (5.4)	111	6 (5.4)
Experienced a SAE by D11	n (%)	111	6 (5.4)	111	7 (6.3)
Experienced a SAE by D21	n (%)	111	6 (5.4)	111	8 (7.2)
Pain score for largest bubo <3 at D4	n (%)	78	30 (38.5)	75	24 (32.0)
Pain score for largest bubo <3 at D11	n (%)	50	31 (62.0)	53	26 (49.1)
Percentage difference in largest bubo size at D4	Median (range)	92	-14.1 (-100.0, 118.3)	94	-12.9 (-100.0, 97.8)
Percentage difference in largest bubo size at D11	Median (range)	92	-46.0 (-100.0, 191.1)	94	-30.1 (-100.0, 160.3)
Percentage of patients who adhered to treatment schedule		111	111 (100)	111	110 (99.1)

N reflects the denominator for each outcome in each arm; for fever N is the number of patients alive with available fever data, for bubo data N is the number of patients with a measurable bubo at relevant time points, for pain score there is no pain measurement for resolved buboes.

Table S13 Risk of bias -- GRADE (Cochrane manual):

**Assessment criteria**	**Assessment outcome**
**Risk of bias**	**Not serious**
Inadequate methods of sequence generation	No
Lack of allocation concealment	No
Lack of blinding of participants	Yes
Lack of blinding of providers	Yes
Lack of blinding of outcome assessors	No
Loss to follow-up	Low
Failure to follow intention to treat principles in analyses	No
Selective outcome reporting of outcomes and/or analyses	No
Other sources of bias	No
**Inconsistency**	**N/A**
**Indirectness**	**Not serious**[1]
**Imprecision**	**Not serious**[2]

[1] tested regimens included in guidelines and used routinely; representative patient population (61% of all cases in the districts where study took place); both sexes and all ages included.

[2] Composite outcome in the primary analysis occurred in <10% of patients, consistent in all analysis populations (ITTI, ITT, PPI, PP), with RD within 1% on either side (favouring control or intervention) and 95% CIs within 9%. The only discrepant analysis is secondary analysis including 25% decrease in bubo size at day 11.

REFERENCES

1. Bourner J, Randriamparany R, Rasoanaivo TF, et al. Bubonic plague: can the size of buboes be accurately and consistently measured with a digital calliper? Trials 2023;24(1):815. (In eng). DOI: 10.1186/s13063-023-07835-7.
